# Pitfalls in Interventional Pain Medicine: Hyponatremia after DDAVP for a Patient with Von Willebrand Disease Undergoing an Epidural Steroid Injection

**DOI:** 10.1155/2017/6467090

**Published:** 2017-03-14

**Authors:** Talal W. Khan, Abdulraheem Yacoub

**Affiliations:** ^1^Department of Anesthesiology and Pain Medicine, University of Kansas Medical Center, Kansas City, KS, USA; ^2^Division of Medical Oncology, Department of Internal Medicine, University of Kansas Medical Center, Kansas City, KS, USA

## Abstract

Desmopressin (DDAVP), a synthetic analog of vasopressin, has been used in patients with von Willebrand disease (VWD), mild hemophilia A, and platelet dysfunction to reduce the risk of bleeding associated with surgical and interventional procedures. We report the case of a patient with VWD presenting with a bulging disc and radicular pain that underwent transforaminal epidural steroid injections. Her course was complicated with the interval development of headaches and dizziness symptomatic of moderate hyponatremia, likely due to excessive fluid intake. This report highlights a relatively rare side effect of DDAVP when used for prophylaxis in patients with VWD and reinforces the need for vigilance in these patients.

## 1. Introduction

Von Willebrand disease (VWD) is the most commonly inherited bleeding disorder with a prevalence of at least 0.1% and a global population of approximately 5.8 million affected individuals [1]. The hallmark of this disease includes a qualitative and/or quantitative deficiency in the most active forms of von Willebrand factor (VWF), which results in abnormal platelet adhesion and relative deficiency of factor VIII. Although the majority of cases present with mild disease, the risk of bleeding is proportional to the degree of deficiency of VWF [2]. VWD is classified into three major categories: type 1 (partial quantitative deficiency), type 2 (qualitative deficiency), and type 3 (severe quantitative deficiency). Type 2 is classified further into four variants based upon phenotype [3].

DDAVP, a synthetic analog of L-arginine vasopressin, is the treatment of choice for the perioperative and periprocedural management of VWD, primarily for type 1 patients who respond to the drug and do not have a contraindication for its use. When administered intravenously, this can cause a rapid increase of up to eightfold in circulating levels of factor VIII and VWF through release of endogenous VWF from endothelial Weibel-Palade bodies [4].

Although neuraxial techniques are commonly avoided in patients with VWD and other bleeding disorders secondary to the risk of hemorrhagic and subsequent neurologic complications, a review by Choi and Brull [5] suggests that even though ideal factor and platelet levels remain undefined for this patient population, the risk of adverse sequelae may be low with appropriate assessment and management of these bleeding diatheses. To our knowledge, only a single abstract reports the management of a lumbar epidural steroid injection in a patient with VWD [6]. The patient reviewed the case report and provided written permission for the authors to publish the report. Both authors participated in the care of the patient described in the case report.

## 2. Case Presentation

The patient was a 44-year-old woman with a lifetime history of low VWF and clinically significant bleeding manifestations. Her disease manifested with excessive bleeding including menorrhagia and epistaxis. Laboratory evaluation confirmed type 1 VWD with ristocetin cofactor activity of 38%, von Willebrand antigen level of 41%, factor VIII level of 60%, and a normal von Willebrand factor multimer distribution. The patient had type A blood and abnormal Platelet Function Assay (PFA) markedly prolonged clotting times with both collagen/epinephrine and collagen/ADP stimulation. The patient previously underwent a DDAVP challenge test which more than doubled her ristocetin activity after administration of 20 mcg of intravenous DDAVP. This challenge test was not followed by any complications. She presented to the pain clinic with a long-standing history of low back and bilateral lower extremity pain. Over the years, she had tried a variety of medications, multiple episodes of physical therapy, and other conservative care without significant improvement. MRI imaging of the lumbar spine revealed an annular tear and disc bulge at L5-S1. After discussion of risks versus benefits of the procedure and the need for consideration of DDAVP to reduce the risk of bleeding, the patient underwent an infusion of DDAVP 0.3 mcg/kg receiving a total of 22 mcg over a period of 30 minutes immediately before the procedure. The patient was not given any specific instructions for fluid restriction postoperatively. Bilateral L5-S1 transforaminal epidural steroid injections were carried out. Once needle position and good contrast spread along the nerve roots were confirmed, an injection of 1 mL of 0.25% bupivacaine and 40 mg methylprednisolone was carried out on each side. Although the procedure was performed without incident, the patient experienced persistent dizziness and a nonpositional headache early the next day. Thorough investigation, including serial serum and urine electrolyte evaluations, revealed findings consistent with hyponatremia (serum sodium: 125 mmol/L), which was a decrease from baseline serum sodium of 138 mmol/L. Further investigation also revealed excessive water intake of 80 ounces per day that may have contributed to the acute onset of hyponatremia. The electrolyte disorder was gradually corrected over a period of 24 hours through fluid restriction and administration of sodium with complete resolution of symptoms. Serum sodium went from 125 mmol/L to 128 mmol/L at the 6th hour after diagnosis, to 134 mmol/L at the 10th hour, and then to 139 mmol/L at the 18th hour after diagnosis and remained at 136–139 mmol/L for the next several days to weeks on outpatient follow-up.

## 3. Discussion

Our case demonstrates the potential for serious complications following the administration of DDAVP for mitigation of bleeding risk in a patient with VWD undergoing an interventional spine procedure. DDAVP results in an increase in VWF activity, factor VIII activity, and plasminogen activation in patients with VWD [7]. DDAVP also results in a potent antidiuretic effect through the impairment of free water absorption by the distal tubules, collecting tubules, and collecting ducts by binding to the arginine vasopressin receptor and reducing water permeability [8]. This antidiuretic hormone effect can persist for several hours after DDAVP clearance. During this period of time, the retention of free water and hyponatremia can occur. Williford and Bernstein [9] reported the occurrence of severe hyponatremia and seizures in children and adults 1 to 4 days after the administration of DDAVP (Table 1).

Certain physiological changes in the periprocedural phase may potentiate the antidiuretic effect of DDAVP. These include an increase in antidiuretic hormone secretion by the posterior pituitary gland in response to physiological stress, pain, and anxiety related to the primary pain condition, as well as the procedure itself. In addition, this particular patient may have been susceptible to free water overload as the patient was in the habit of drinking over 80 ounces of water per day (Table 2). In addition to excessive water intake that contributed to the symptomatic hyponatremia, the patient also received a dose of DDAVP exceeding 20 mcg. Despite the relatively short half-life of DDAVP of 2 hours, the risk of water intoxication and hyponatremia persisted for several hours after the procedure. This patient also had a history of epilepsy, which was of concern. The patient did not present any epileptic events as a complication but did require hospitalization for observation for 24 hours.

The nature of the disorder of hemostasis, resting factor levels, target factor levels, and the length of time required for maintenance of factor levels for any given procedure dictate the clinical indications for use of DDAVP. When used judiciously, this agent has few troublesome side effects ranging from mild facial flushing to tachycardia and transient headaches. If excessive fluid intake is avoided, signs of more serious hyponatremia and cerebral edema are rare [11]. The authors recommend a thorough discussion of risks versus benefits and alternatives with patients as well as education regarding signs and symptoms that might suggest a more serious problem. Fluids must be restricted to maintenance for 24 hours after any dose of DDAVP. In general, more cautious dosing of DDAVP is recommended. It has been found that sodium nadir occurs within 9–20 hours of DDAVP administration [12]. The healthcare provider, patient, and family should be urged to maintain a high level of vigilance for symptoms suggesting hyponatremia and, if evident, the patient should seek medical treatment promptly to avoid life-threatening complications.

## Figures and Tables

**Table 1:** Clinical manifestations of hyponatremia (Na < 135 mmol/L).

Nausea
Vomiting
Headache
Lethargy
Malaise
Muscle cramps
Restlessness
Disorientation/confusion
Depressed reflexes
Seizures
Coma
Respiratory arrest
Brain stem herniation
Irreversible brain damage
Death

**Table 2:** Risk factors for hyponatremia after DDAVP [10].

Stress
Surgery
Administration of anesthesia
Opiates (endogenous release of ADH)
Vomiting (loss of sodium)
Liver disease (impaired metabolism of DDAVP)
Renal tubular acidosis (chronically low sodium)
Multiple doses of DDAVP
Overhydration with hyponatremic fluids
